# Septic arthritis following femoral neck fracture: A case report

**DOI:** 10.1016/j.ijscr.2019.03.016

**Published:** 2019-04-04

**Authors:** Chulin Chewakidakarn, Anuchit Nawatthakul, Methasit Suksintharanon, Varah Yuenyongviwat

**Affiliations:** Department of Orthopedics, Faculty of Medicine, Prince of Songkla University, Songkhla, 90110, Thailand

**Keywords:** Femoral neck fracture, Septic arthritis, Total hip replacement, Case report

## Abstract

**Introduction:**

Femoral neck fracture is common in the elderly, especially in the era of aging populations. Septic arthritis of the hip after femoral neck fracture is a rare complication. There are limited reports on this complication especially in patients without a severe medical condition and there is no consensus on the method of treatment of this complication.

**Case presentation:**

A 75-year-old male had right hip pain for two weeks after falling on his right hip. The patient was diagnosed as pathological fracture with hematologic malignancy. He was scheduled for bipolar hemiarthroplasty. However, an intraoperative finding found that the hip joint was filled with pus. The treatment was successful with a two-stage procedure. The first stage was implantation of an antibiotic cement spacer and the second stage was total hip replacement.

**Conclusion:**

Septic arthritis following femoral neck fracture is a complication which requires special attention for the diagnosis and treatment. Increasing hip pain or the developing signs of infection in patients awaiting surgery may indicate infection in the hip. The two-stage procedure is the treatment of choice in this condition.

## Highlights

• Septic arthritis following femoral neck fracture is a complication that requires special attention for diagnosis.
• The surgeon should perform definite treatment for femoral neck fracture as soon as possible.
• The two-stage procedure is the treatment of choice in septic arthritis following femoral neck fracture.

## 1 Introduction

In the era of aging populations, femoral neck fracture is a common problem in the elderly. The world-wide incidence was 1.66 million in 1990 and might increase to 6.26 million in 2050 [1]. The mortality and morbidity of hip fracture are high with one-year mortality rates that range 14–36% [2]. Surgery is the treatment of choice if there is no medical contraindication for surgery such as recent myocardial infarction or recent stroke. Non-operative treatment with bed rest is associated with problems of immobilization, such as pressure sore, pneumonia, and urinary tract infection, which increase the mortality 3.8 times in comparison with the operative group [3]. Septic arthritis of the hip after femoral neck fracture is a rare complication. There were reports of a few cases of septic arthritis in patients following femoral neck fracture with severe medical comorbidities [4,5]. However, there are no reports of this complication in patients without severe medical conditions and there is no consensus on the method of treatment of this condition. This report was made according to the SCARE criteria [6].

## 2 Presentation of case

A 75-year-old male was referred from another hospital with right hip pain and the inability to walk. Two weeks prior to this admission, he fell onto his right hip from a standing position. After that incident, he developed right hip pain but could walk with support. He was treated as contusion with nonsteroidal anti-inflammatory drugs for pain relief at a clinic. One day prior to referral to the authors’ hospital, he developed sudden right hip pain during a twisting motion of his right hip without slipping or falling. He could not tolerate weight bearing on his right leg and thus was taken to a nearby hospital. The patient was diagnosed with pathological fracture of the femoral neck from hematologic malignancy and was referred to the authors’ hospital.

**Fig. 1:**
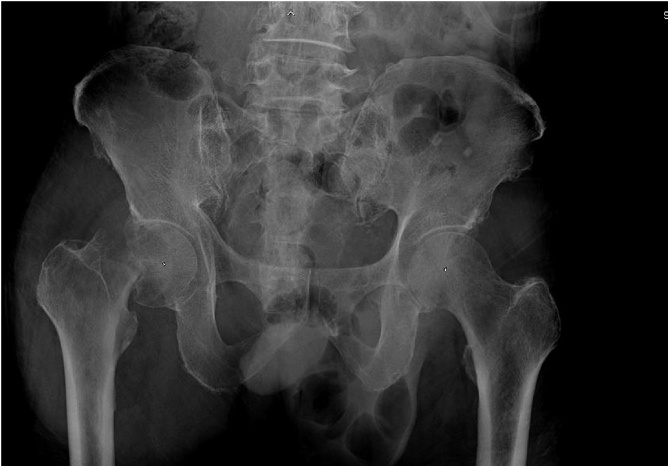
Initial plain radiograph of fracture.

**Fig. 2:**
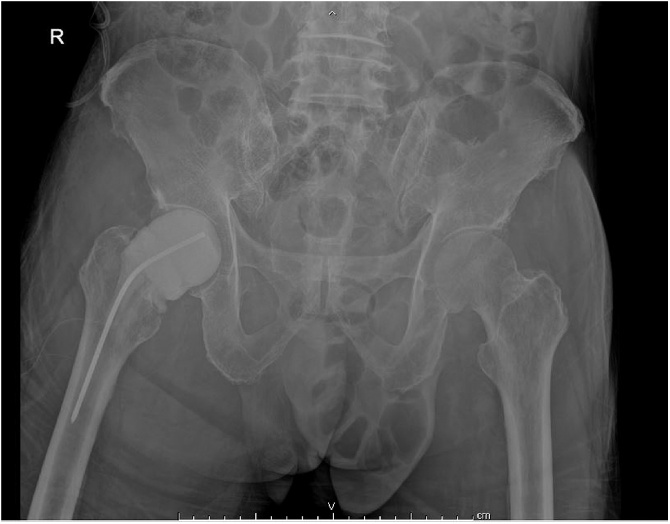
Plain radiograph after the first operation.

Upon admission the patient was afebrile and the physical examination showed no signs of inflammation or skin lesion. A plain radiograph showed subcapital fracture of the right femoral neck with osteolytic lesions (Fig. 1). The results of the initial serum workup were white blood cell count 19,490/μL, hematocrit 26.7%, hemoglobin 8.6 g/dL, polymorphonuclear neutrophils (PMN) 85.5%, lymphocytes 6.0%, eosinophils 0.2%, monocytes 8.2%, and platelets 336,000/μL. The erythrocyte sedimentation rate (ESR) was more than 140 mm/h (normal value 0–15 mm/h) and the C-reactive protein (CRP) was more than 19.2 mg/dL (normal value <0.6 mg/dL). He was scheduled for bipolar hemiarthroplasty. However, an unexpected intraoperative finding completely changed the surgical plan. His right hip joint was filled with about 20 mL of frank pus. The operation was shifted to irrigation and debridement. The femoral head and neck were sent for tissue study. Necrotic tissue and synovial tissue were removed. The wound was then copiously irrigated with 6 liters of normal saline. An antibiotic cement spacer was prepared with two packs of premixed-gentamycin bone cement (Refobacin® Bone Cement R; Zimmer Biomet, Warsaw, IN, USA) mixed with 6 g of vancomycin powder (Vancogen; Alkem Laboratories Ltd, Daman, India). A cement spacer was inserted to function as a temporary hemiarthroplasty (Fig. 2).

Cultures of the debrided tissue and pus from the operation grew *Staphylococcus aureus*. On the day after the operation, a hemoculture which was drawn from the patient prior to admission grew *Staphylococcus aureus.* A pathological tissue diagnosis reported acute osteomyelitis with abscess at the head and neck of the femur.

**Fig. 3:**
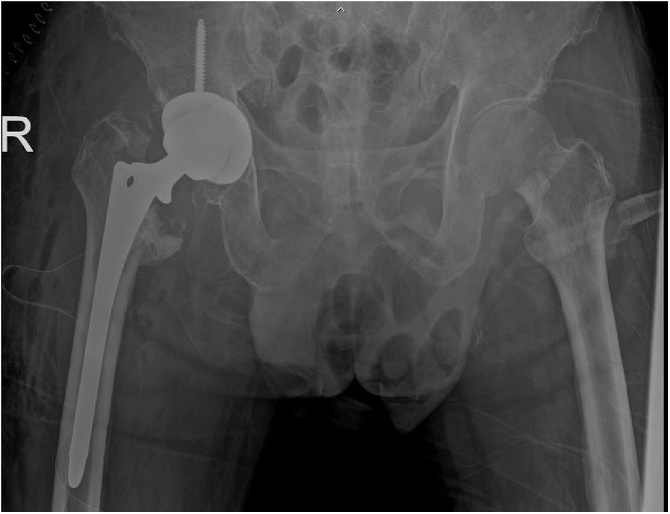
Immediate post-operative plain radiographs after revision total hip replacement.

**Fig. 4:**
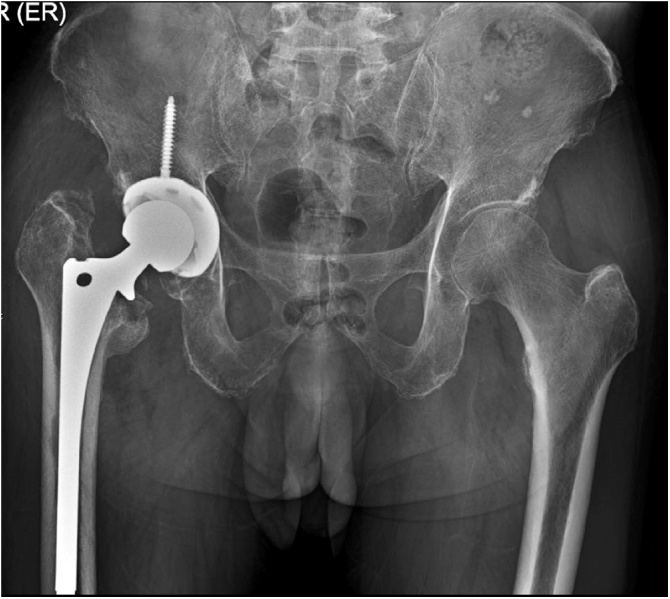
Plain radiographs at 6 months after revision total hip replacement.

Post-operatively, the patient received ceftriaxone and clindamycin intravenously as empirical treatment for three days and then later switched to cefazolin intravenously according to the culture and sensitivity results. The total duration for intravenous antibiotics was two weeks. The patient then received oral dicloxacillin for two months and cephalexin for four months. His clinical condition was good and the inflammation markedly decreased (ESR 59 mm/h, CRP < 0.6 mg/dL). Antibiotic treatment was discontinued without relapse of symptoms and no elevation of inflammatory markers. Five weeks later, he underwent revision total hip arthroplasty with a cementless prosthesis. His initial post-operative revision hip arthroplasty radiographs are shown in Fig. 3. After surgery, he was able to walk with full weight bearing assisted by single cane support. Upon the latest follow-up visit at six months post-operatively, his general condition was good and his Harris hip score was 75. The plain radiograph of his hips is shown in Fig. 4.

## 3 Discussion

Femoral neck fracture is a common problem among elderly patients. This fracture is associated with various complications, high morbidity, and mortality. Early operative treatment, either with internal fixation or hip replacement to gain early ambulation, can minimize these problems [2].

Septic arthritis after femoral neck fracture is a very rare complication that was reported only twice. Colak et al. reported three cases and Hearth et al. reported two cases. All cases in both reports were immunocompromised hosts and the ages varied from 48 to 96 years old [4,5]. Our patient was a 76-year-old male with well-controlled hypertension. Most of the cases in the previous reports had definite surgery at least two weeks after the initial injury which was in the same period as our case that developed septic arthritis around two weeks after the fracture [4,5].

The hypothesis of septic arthritis after hip fracture in this case was hemarthrosis in the hip joint after fracture that was the media for microorganism growth and infection [4]. Local or systemic infection may be identified as the source of the pathogen. The cases in the Hearth et al. report had urinary tract infections as the source of infection. However, in our case the source of infection could not be identified [4].

The elevated white blood cell count, increased percentage of PMNs, and the highly elevated inflammatory markers (ESR and CRP) were found in our case and in the previous case reports [4,5]. The pathogens identified from pus specimens in the previous studies were gram negative, and in most cases the organisms were *Escherichia coli*, *Proteus* species, and *Pseudomonas* species [4,5]. Only one case in the literature was *Staphylococcus aureus* which was similar to our case [5].

There is no specific treatment for this condition because this kind of incident is rare. Hearth et al. performed resection arthroplasty [4]. Colak et al. reported a two-stage procedure for treatment of this problem; however, two cases died during treatment [5]. We considered the two-stage procedure based on the suggested treatment of primary septic arthritis of the hip without fracture in previous reports [7,8]. Total hip arthroplasty was chosen in our patient instead of hemi-hip replacement due to the infection and the prolonged implantation of the cement spacer between the first and second stages which possibly affected the acetabular articular cartilage.

Treatment by the one-stage procedure performed by debridement followed by replacement with a new prosthesis in the same operation was an option for treatment in infected total hip arthroplasty with a good outcome [9]. However, there is limited evidence of this method for treatment in patients with active septic arthritis. Further studies of the one-stage procedure in patients with active septic arthritis may confirm that the one-stage procedure can be used.

## 4 Conclusion

Septic arthritis following femoral neck fracture is a complication which requires special attention for diagnosis. This complication would not be confusing with a pathological fracture from malignancy because the plan for treatment would be completely different. Increasing hip pain or developing signs of infection in a patient awaiting surgery may raise the suspicion of infection in the hip. In another aspect of this condition, the surgeon should perform definite treatment for femoral neck fracture as soon as possible, especially in immunocompromised host patients. Furthermore, the two-stage procedure is the treatment of choice in this condition.

## Conflicts of interest

No conflicts of interest.

## Funding

This case report was funded by the Faculty of Medicine, Prince of Songkla University, Songkhla, Thailand.

## Ethical approval

The present study was approved by the Prince of Songkla University Institutional Review Board, Faculty of Medicine, Songklanagarind Hospital, Prince of Songkla University (IRB number REC 61-414-11-1).

## Consent

Written informed consent was obtained from the patient for publication of this case report and accompanying images. A copy of the written consent is available for review by the Editor-in-Chief of this journal on request

## Author contribution

Chulin Chewakidakarn —Preparation of case report, Literature review, Writing the paper.

Methasit Suksintharanon —Preparation of case report. Writing the paper.

Anuchit Nawatthakul — Preparation of case report. Writing the paper.

Varah Yuenyongviwat—Preparation of case report, Literature review, Writing the paper.

## Registration of research studies

None.

## Guarantor

Varah Yuenyongviwat, MD.
